# Current Advancements in Anti-Cancer Chimeric Antigen Receptor T Cell Immunotherapy and How Nanotechnology May Change the Game

**DOI:** 10.3390/ijms25105361

**Published:** 2024-05-14

**Authors:** Kimberly S. Ku, Jie Tang, Yuan Chen, Yihui Shi

**Affiliations:** 1 College of Medicine, California Northstate University, Elk Grove, CA 95757, USA; kimberly.ku9614@cnsu.edu (K.S.K.); jie.tang9134@cnsu.edu (J.T.); 2 Section Pathology of the Institute of Forensic Medicine, Jena University Hospital, Friedrich Schiller University Jena, Am Klinikum 1, 07747 Jena, Germany; yuan.chen@med.uni-jena.de; 3 California Pacific Medical Center Research Institute, Sutter Bay Hospitals, San Francisco, CA 94107, USA

**Keywords:** immunotherapy, nanomedicine, cancer, CAR-T

## Abstract

Chimeric antigen receptor (CAR)-T cell immunotherapy represents a cutting-edge advancement in the landscape of cancer treatment. This innovative therapy has shown exceptional promise in targeting and eradicating malignant tumors, specifically leukemias and lymphomas. However, despite its groundbreaking successes, (CAR)-T cell therapy is not without its challenges. These challenges, particularly pronounced in the treatment of solid tumors, include but are not limited to, the selection of appropriate tumor antigens, managing therapy-related toxicity, overcoming T-cell exhaustion, and addressing the substantial financial costs associated with treatment. Nanomedicine, an interdisciplinary field that merges nanotechnology with medical science, offers novel strategies that could potentially address these limitations. Its application in cancer treatment has already led to significant advancements, including improved specificity in drug targeting, advancements in cancer diagnostics, enhanced imaging techniques, and strategies for long-term cancer prevention. The integration of nanomedicine with (CAR)-T cell therapy could revolutionize the treatment landscape by enhancing the delivery of genes in (CAR)-T cell engineering, reducing systemic toxicity, and alleviating the immunosuppressive effects within the tumor microenvironment. This review aims to explore how far (CAR)-T cell immunotherapy has come alone, and how nanomedicine could strengthen it into the future. Additionally, the review will examine strategies to limit the off-target effects and systemic toxicity associated with (CAR)-T cell therapy, potentially enhancing patient tolerance and treatment outcomes.

## 1. Introduction

Immunotherapy in cancer treatment is a rapidly advancing field that harnesses the patient’s own innate and adaptive immune system to effectively target tumors whilst minimizing side effects often associated with systemic therapies. Current established methods include the use of monoclonal antibodies, T cell-based therapies, and others to target or regulate various aspects of cancer [1]. Chimeric antigen receptor (CAR)-T cell therapies, especially, have provided a successful avenue through which to target cancer. However, despite the vast progress made in the field, there still exist some shortcomings in cancer immunotherapy. Many of these pertain to immune-related toxicities, limited response rates, and high costs. To assuage these barriers, a variety of strategies have been employed. One of the most notable has been the application of nanotechnology due to its demonstrated ability to block immunosuppressive molecules, reduce toxicity, and increase the anti-tumor effects of T cells at an affordable cost [2,3]. In this review, we will use current literature to explore the various ways in which (CAR)-T cell therapy has made strides in the treatment of cancer and look at the future of nanomedicine integration into this immunotherapy.

## 2. Current Status of (CAR)-T Cell Therapy

**Figure 1:**
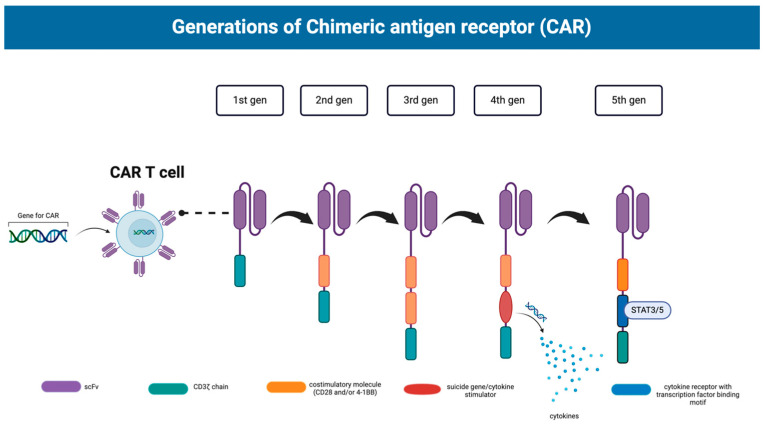
The five generations of CARs contain different elements that increase the efficacy of (CAR)-T cell therapy with each subsequent progression. Each generation consecutively adds more incorporated elements that increase the action, invasion, and longevity of its (CAR)-T cell effects. Created with BioRender.com.

Chimeric Antigen Receptor (CAR)-T cell therapy is a novel therapy that uses manufactured synthetic receptors to promote immune attack of specific target antigens by T cells and other lymphocytes [4]. Their unique ability to bind to target antigens in place of the endogenous T cell receptor and induce T cell activation independent of MHC receptors has been particularly useful in the treatment of cancers due to the unique antigens expressed in cancer cells as well as the components of the tumor microenvironment [5,6]. The structure of the CAR is composed of an extracellular antigen-binding domain, a hinge region, a transmembrane domain, and intracellular signaling domains. The antigen-binding domain is created from monoclonal antibody heavy (VH) and light (VL) chains that connect to form a single-chain variable fragment (scFv) that recognizes both surface and intracellular cancer antigens [4,7]. This antigen-binding domain is significant in that its positioning impacts the affinity and specificity of the CAR for its target antigen [8]. The hinge region is an extracellular region that provides distance between the binding units and the transmembrane region to allow for appropriate immune synapse formation [9]. It affects the flexibility, expression, signaling, and affinity of the CAR [10]. The transmembrane domain mainly functions as an anchor for the CAR, although some studies illustrate that it can affect CAR expression, stability, and signaling [4]. More importantly, the hinge region and transmembrane region together have been shown to impact (CAR)-T cell cytokine production and activation-induced cell death [11]. The intracellular signaling domains, however, have received the most attention in optimizing CARs. Variations in these domains have produced four generations of CARs (Figure 1): the first containing just a CD3ζ chain, the second containing a CD3ζ as well as costimulatory molecules such as CD28 and 4-1BB, the third containing both CD28 and 4-1BB costimulatory molecules in a paired fashion, and the fourth containing these as well as cytokines, chemokine receptors, and a suicide gene [12,13]. Very recently, a fifth generation of (CAR)-T cells has been introduced. This generation comprises an extra intracellular domain of cytokine receptors in comparison to their predecessors with a motif to bind transcription factors such as STAT3/5 [12]. This allows for not only the activation of T cells and the production of memory cells but also the stimulation of the immune system [14].

### 2.1. Hematological Malignancies

One of the greatest successes that (CAR)-T cell therapy has seen is in the scope of hematological malignancies, with applications in acute lymphoblastic leukemia (ALL), chronic lymphocytic leukemia (CLL), and multiple myeloma. These cancers provide an easier target for CARs due to their adequate tumor antigens, most prominently CD19 [15]. As of 2020, (CAR)-T cell therapies accounted for over half of developing cell therapies on the market, with 858 (CAR)-T cell therapies among a total of 1483 anti-cancer cell therapies [16,17]. The overwhelming triumph that (CAR)-T cell therapy has shown in the treatment of B-cell malignancies in particular has led to the Food and Drug Administration (FDA) approving this unique therapy, consequently encouraging further exploration of this cancer treatment strategy [18,19,20]. Kymriah, a (CAR)-T produced by Novartis, has illustrated significant effects on B-ALL, with an 81% overall remission rate within 3 months [19,21]. Yescarta (axicabtagene ciloleucel) has also been approved by the FDA, with 58% complete responders and 25% partial responders for the treatment of refractory large B-cell lymphomas [22]. Breyanzi (lisocabtagene marleucel) has been approved for the same [23]. Abecma (idecabtagene vicleucel) has been approved as the first anti-BCMA (CAR)-T cell therapy for the treatment of refractory multiple myeloma. The current FDA-approved (CAR)-T therapies are summarized in Table 1.

Despite these promising developments, some difficulties still exist in cancers like CLL and ALL due to factors not limited to immune system dysregulation and the absence of targeted antigens [24]. Solid tumors have provided an even greater challenge to the clinical application of (CAR)-T cell therapy.

**Table 1:** Current FDA-approved (CAR)-T cell therapies [17,23,25,26,27,28].

Generic Name	Brand Name	Target	Year of Approval	Disease
Tisagenlecleucel	Kymriah^®^	CD19	2017	Acute lymphocytic leukemia
Axicabtagene	Yescarta^®^	CD19	2017	Diffuse large B-cell lymphoma
Brexucabtagene autoleucel	Tecartus^®^	CD19	2020	Mantle cell lymphoma
Lisocabtagene maraleuce	Breyanzi^®^	CD19	2022	Adult large B-cell lymphoma
Ciltacabtagene autoleucel	Abecma^®^	B-cell maturation antigen (BCMA)	2022	Multiple myeloma
Ciltacabtagene autoleucel	Carvykti^®^	BCMA	2022	Relapsed or refractory Multiple myeloma

### 2.2. Solid Tumors

The application of (CAR)-T cell therapy in blood cancers has demonstrated promising results, encouraging researchers and clinicians to apply this therapeutic strategy in the treatment of solid tumors. Indeed, several clinical trials using (CAR)-T cell therapy in different types of solid tumors, including carcinoma and sarcoma, are currently ongoing [29]. These (CAR)-T cell-based clinical trials target antigens such as mesothelin (MSLN), epidermal growth factor receptor (EGFR), disialoganglioside (GD2), HER2, mucin 1 (MUC1), carcinoembryonic antigen (CEA), EpCAM, etc., for the treatment of solid tumors such as colorectal carcinoma (CRC), ovarian cancer, retinoblastoma, lung cancer, breast cancer, gastric cancer, and prostate cancer (Table 2) [30]. In fact, several of these have entered phase I/II trials and demonstrated encouraging outcomes [30]. A phase I escalating-dose trial of (CAR)-T therapy targeting CEA-positive metastatic CRC, (CAR)-T cells at five dose levels (from 1 × 10^5^ to 1 × 10^8^ CAR/kg cells) has displayed safety and efficacy of treatment with tumor reduction observed in 7 out of 10 patients after (CAR)-T cell therapy [31]. (CAR)-T cells combined with chemotherapy or PD-1/PD-L1 blockade have also been widely used in preclinical models or clinical trials for the treatment of solid tumors. Evidence shows that chemotherapy can deplete immunosuppressive cells, promote a pro-inflammatory tumor microenvironment, disrupt tumor stroma, and improve (CAR)-T cell recruitment to the tumor [32]. In a recent study, the chemotherapy drugs Paclitaxel, Cyclophosphamide, and Fludarabine were used as an adjuvant regimen combined with Claudin18.1-(CAR)-T cells for the treatment of patients with gastric cancer. It was found that 21 out of 28 patients who had previously failed taxane treatment achieved therapy responses with this regimen [33]. In prostate cancer, adoptive hPSMA-(CAR)-T cell immunotherapy was enhanced when combined with PD-1 blockade in vitro and in vivo [34]. This combination therapy may provide groundbreaking treatment for cancer patients henceforth. However, challenges remain in the use of (CAR)-T cell therapy for solid tumors. There have been many difficulties in applying the same strategy of hematological malignancies to solid tumors due to the lack of effective antigen targets and the existence of suppressive cytokines (TGF-β, IL-10), tumor-associated macrophages (TAMs), regulatory T cells (Tregs), and myeloid-derived suppressor cells (MDSCs) as well as hypoxia and high pressure, which limit access to the microenvironments of solid tumors [35]. These characteristics lead to exacerbation of the problems already inherent in T cell therapy and ultimately interfere with (CAR)-T cells’ ability to recognize tumor antigens and invade the tumor [36]. 

Nonetheless, there has been an increasing number of trials that aim to develop methods by which we can circumvent these obstacles, whether by producing multi-specific CARS, expressing tumor-specific factors on the surface of T cells, or using neutralizing antibodies to block cytokines and reverse T cell inhibition (Table 3) [37]. Muhammad et al. have addressed the issue of suitable tumor targets with a newly developed tandem CAR (TanCAR) that targets both ephrin type-A receptor 2 (EphA2) and interleukin-13 receptor subunit α-2 (IL-13Rα2) in glioblastoma (GBM) models. In preclinical studies, these TanCARs were found to reduce gliomas with more efficacy than single-target CARs and potentially prevent antigen escape and off-target cytotoxicity [38]. Another multi-target CAR targeting GD2, CD44v6, and HER2 is currently in clinical trials for the treatment of breast cancer (NCT04430595). Liu et al. looked to exploit the specific chemokines and cytokines in the TME by developing a GDPC3 CAR modified to express CXCR2, which is highly expressed in human hepatocellular carcinoma (HCC). In their xenograft tumor model, they found that this modification significantly increased the migration and accumulation of the CAR-T cell into the tumor [39]. Tumor markers such as fibroblast activating protein (FAP) have been targeted as well due to their remodeling of the TME and subsequent prevention of T cell invasion. A fourth generation (CAR)-T cell therapy that targets Nectin4/FAP is currently in Phase I clinical trials for the treatment of advanced malignant solid tumors (NCT03932565). As for the immunosuppressive and hostile environment of the tumor, several targets have been studied to allow for better (CAR)-T cell action. Programmed cell death protein-1 (PD-1) continues to be of particular interest as PD-1 inhibition along with (CAR)-T cell therapy has shown greater efficacy both preclinically and clinically [40]. A MUC-1-targeted (CAR)-T cell therapy with PD-1 knockout is currently in clinical trials for the treatment of non-small cell lung cancer (NCT03525782). For issues with the hypoxic environment of the TME, hypoxia-inducible CARs (HiCARs) have been developed that include oxygen-dependent degradation domains (ODD) as well as hypoxia response elements (HRE) to maintain stability in low-oxygen environments such as the TME [41].

**Table 2:** Current (CAR)-T cell therapy targets in solid tumors. Many of these targets are found in a vast variety of tumor types, but the listed include those that have been included in published studies [33,42,43,44,45,46,47,48,49,50,51,52,53,54].

Target Antigen	Tumor Type
EGFRvIII	GB, NSCLC
IL13Ralpha2	GB
HLA-G	CRC, GC, OC, thyroid cancer, cervical cancer, endometrial cancer [43]
Mesothelin	MPM, PDAC, OC
HER2	GB, pancreatic, bile duct
PSMA	PCa
Mucin-1	Pancreatic, NSCLC
GD2	Osteosarcoma, neuroblastoma, glioma
NKG2D	CRC
Claudin18.2 (CLDN18.2)	GC
CEA	CRC, other CEA-positive tumors
EpCAM	pancreatic
GPC3	HCC
B7H3	CNS malignancies and others
FAP	CRC, OC, lung cancer, PDAC

GB: glioblastoma, NSCLC: non-small cell lung cancer, MPM: malignant pleural mesothelioma, PDAC: pancreatic ductal adenocarcinoma, OC: ovarian cancer, CRC: colorectal cancer, HCC: hepatocellular carcinoma, GC: gastric cancer, PCa: prostate cancer.

**Table 3:** Currently active phase II clinical trials for (CAR)-T cell therapy in adult solid tumors. Suspended and terminated studies have not been included.

Clinical Trial	Target	Status
NCT04348643	CEA	Recruiting
NCT06006390	CEA	Recruiting
NCT05538195	CEA	Recruiting
NCT05947487	CD70	Recruiting
NCT05944185	MSLN	Not yet recruiting
NCT03179007	MUC1	Unknown
NCT05748938	ROR1	Recruiting
NCT03182816	EGFR	Unknown
NCT05812326	MUC1	Completed
NCT03706326	MUC1	Unknown
NCT03030001	Mesothelin	Unknown
NCT03356795	GD2, PSMA, Muc1, Mesothelin	Unknown
NCT05693844	MSLN	Recruiting
NCT03182803	Mesothelin	Unknown
NCT05437315	GD2/PSMA	Recruiting
NCT03356782	CD133, GD2, Muc1, CD117, and other sarcoma markers	Unknown
NCT05736731	CEA	Recruiting
NCT06051695	MSLN	Recruiting
NCT03615313	Mesothelin	Unknown
NCT03373097	GD2	Recruiting
NCT04581473	CLDN18.2	Recruiting
NCT06150885	HLA-G	Not yet recruiting
NCT05120271	GPC3	Recruiting
NCT02873390	EGFR	Unknown
NCT02862028	EGFR	Unknown
NCT02830724	CD70	Recruiting
NCT02992210	GD2	Unknown
NCT06082557	TROP2	Not yet recruiting

### 2.3. Limitations

One of the most important aspects of (CAR)-T cell therapy is the selection of an appropriate tumor antigen to target. Without this, there is nothing for the T cell to recognize and attack. This brings us to one of the ways by which cancer resists this therapy: tumor escape. Tumor escape occurs because of cancer cells’ ability to modify their antigens and become resistant to the CARs directed at those antigens. Some mechanisms include production of immunosuppressive molecules such as IL-10, TGF-β, and indoleamine 2,3-dioxygenase (IDO), overexpression of immunosuppressive receptors like PD-L1, altered expression of G1 regulatory proteins, and stimulation of Treg activity [55]. These abilities of the tumor cells and the tumor microenvironment prevent (CAR)-T cell therapy from maximizing its potential efficacy. 

Another limitation of (CAR)-T cell therapy is the toxicity that may be associated with (CAR)-T cells. This includes cytokine release syndrome (CRS), neurotoxicity, and on-target-off-tumor toxicity. CRS is defined as the release of a massive amount of inflammatory molecules (IL-1, IL-6) by (CAR)-T-cells that subsequently cause chronic fever, dyspnea, hypotension, and organ damage [56]. It is notable that patients with large lesions, high tumor burden, complications, or initiation of CRS 3 days after (CAR)-T cell therapy have a higher risk for severe CRS [57]. Neurotoxicity typically presents as confusion, decreased consciousness, seizures, and brain edema that may occur with or without CRS. On-target-off-tumor toxicity is associated with the antigens targeted by (CAR)-T cell therapy. Ideally, the tumor antigen would be expressed only on cancer cells, but this is unfortunately not the case and mistargeting is highly possible. This leads to toxicity in normal tissues that may express the same antigen that the tumor target does [58].

The last limitation of this immunotherapy is T cell exhaustion defined by T cell dysfunction that develops in many chronic diseases. It is characterized by decreased effector function and sustained expression of inhibitory receptors. These T cells are transcriptionally distinct from a typical effector or memory T cell [59]. There are many genes responsible for the regulation of T cell exhaustion, including the NR4A family (nuclear receptor transcription factors), which have provided targets for the prevention of T cell exhaustion associated with this anti-cancer therapy [60].

Outside of the physiological limitations of this therapy, there is also a financial burden associated with the highly specific nature of the cell-based therapy due to the necessity of engineering receptors for (CAR)-T tumor antigens [61]. This multitude of limitations has led to further developments of (CAR)-T cell therapy to assuage some of the shortcomings of this immunotherapy. These include the advent of universal (CAR)-T cell therapy, which consists of allogeneic (CAR)-T cells from healthy donors rather than autologous T cells [16]. However, we will focus here on the introduction of nanoengineering, which has illustrated an ability to mitigate the many barriers we have discussed and has helped provide an exciting new avenue for improvement of our current anti-cancer treatments.

## 3. Incorporation of Nanotechnology into (CAR)-T Cell Therapy

With the numerous barriers we already discussed that provide a challenge to accessible and effective T cell therapy, nanomedicine and its adaptable nature provides a potential solution to a number of these problems, given their contributions to other aspects of cancer care [62].

### 3.1. T Cell Engineering

**Figure 2:**
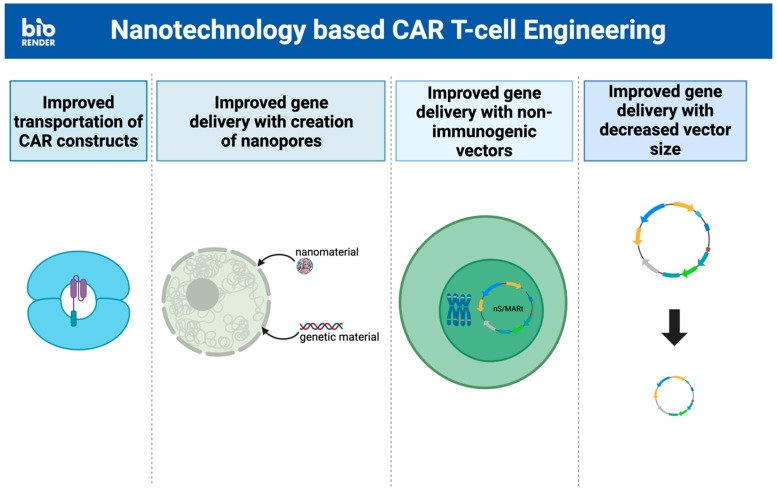
Examples of improvement in (CAR)-T cell engineering via incorporation of nanotechnology. Improved delivery of CAR constructs and mRNA can be seen with the use of AuNPs and liposomes. Ease of transportation into T cells has been achieved with nanopores, specialized plasmid vectors, and nanoplasmids. These strategies all help with easing the burden of creating specialized CARs. Created with Biorender.com.

The challenges involved in T cell therapy begin with the engineering of (CAR)-T cells. The delivery of the engineered CAR constructs can be difficult due to mechanisms of resistance such as opposition to passage through plasma cell membranes. Traditionally, viral vectors have been used to overcome this obstacle, but these vectors have their own problems of cargo limitations, biological safety, and production efficiency [63]. These obstacles have led to the exploration of nanoscale technologies as a method of CAR gene delivery to effectively bypass the plasma membrane and minimally damage the cell (Figure 2).

The gold nanoparticle (AuNP) has traditionally been used as a contrast agent in biomedical imaging due to its unique optical property, known as surface plasmon resonance, and its ability to penetrate tumor/cellular tissues [45]. Additionally, AuNP’s non-immunogenic nature as a noble metal and its capability for complete elimination through the glomerular filtration system has shown great potential as an effective and non-toxic tool to be combined with immunotherapy [46]. Its capacity to be quickly synthesized with high yield using the citrate reduction method only promotes its use in all aspects of cancer therapy. Shokohui et al. demonstrated how these virtues can be ingeniously applied to the application of immunotherapy by engineering a scalable and reusable electroactive nano-injection platform that effectively delivers anti-CD19 CAR constructs into human T cells while maintaining the viability of these cells. This platform is based on Au-coated NT arrays with a cavity that allows for direct cargo loading. The engineered (CAR)-T cells have already demonstrated anti-proliferative effects on Raji lymphoma cells in vitro [64]. 

Liposome nanocarriers have also shown great potential due to their lipid bilayer composition, which is similar to the cellular membrane. They provide major advantages in terms of biocompatibility and the ability to encapsulate hydrophilic, lipophilic, and amphiphilic compounds to prevent their toxic effects [52]. Additionally, the highly modifiable surface of liposome vesicles can be modified with targeting ligands, antibodies, and peptides for potential targeted delivery [55]. Kitte et al. have leashed the potential of liposomes to improve the process of engineering (CAR)-T cells in their study comparing lipid nanoparticles (LNPs) to established electroporation (EP) techniques for ex vivo CAR-mRNA delivery. In their study, LNPs were loaded with mRNA to produce mRNA-based (CAR)-T cells. These LNP–(CAR)-T cells showed greater longevity in CAR-mRNA persistence and expression, allowing for greater efficacy, which was attributed to the lower cytotoxicity and slower (CAR)-T cell proliferation of the LNP-aided technique [65].

Hur et al. demonstrated the efficacy of another stable and scalable T cell nanoengineering system that creates nanopores in T cells. This system utilizes cell mechanoporation via microchannel geometry and hydrodynamic flows to efficiently deliver nano molecules and other biomolecules into human primary T cells without altering their gene expression [48]. Initially, CD3+ T cells are mixed with a medium containing functional nanomaterials intended for delivery into the T cell. This cell suspension is then injected into a microfluidic channel comprising one core channel for cell injection, two sheath channels, and two sheath inlets to position the T cell at the channel center using inertial and hydrodynamic forces. As the T cells approach the stagnation point of the channels, where the flow rate significantly slows down, they undergo transient physical elongation and restoration. This transient elongation creates pores within the cell membrane, facilitating the internalization of nanomolecules or other biomolecules in the cell suspension. In this study, the internalization of fluorescein isothiocyanate (FITC)-dextran into primary T cells was utilized to assess the efficacy of the cell mechanoporation system. The successful internalization of FITC-dextran, which is approximately 54 nm in diameter, indicates that larger nanomaterials such as gold and polymeric nanoparticles can also be delivered into primary T cells using this method. Furthermore, the scalability of this approach was tested by varying the concentration of T cells, demonstrating that up to 1 million T cells can be processed within 1 min using a single channel without reducing delivery efficiency [66]. 

Another option for nanotechnology-reliant gene delivery is the nano-S/MARt (nS-MARt) plasmid vector described by Bozza et al. This novel DNA vector platform can replicate extrachromosomally in nuclei and allow for the maintenance of T cell transgene expression without negatively affecting human T cells. Additionally, its lack of immunogenic components avoids problems that are traditionally seen in viral vectors [67]. An even simpler method of incorporating nanotechnology into gene delivery for (CAR)-T cell engineering is using them to decrease vector size. The transposition of transposon-engineered anti-BCMA (CAR)-T cells, in particular, is improved with the incorporation of nanoplasmids. This manufacturing change had the dual advantage of decreasing backbone size and decreasing distance between inverted terminal repeats (ITRs), ultimately resulting in high amounts of T stem cell memory (TSCM) in patients [68]. 

Lastly, the use of nanomaterials for gene delivery has the advantage of being much more cost-effective. This is due to the simple production process that allows for the production of CAR monocytes in as little as 1 day, greatly reducing overall treatment time and cost [69].

### 3.2. Avoiding Systemic Toxicity

(CAR)-T cell therapy is associated with numerous toxicities due to its systemic quality. Because of this, some studies have evaluated the efficacy of local immunotherapy in cancer treatment. They have observed that even local injection of immune cells or immunostimulatory molecules can provide systemic anti-cancer immunity while preventing the widespread toxicity associated with many systemic immunotherapies. Although this has mainly been observed with CD40 agonistic antibodies, this provides an interesting avenue through which the neurotoxicity and CRS of (CAR)-T cell therapy might be resolved, especially in the treatment of solid tumors [70,71,72]. Additionally, on-tumor off-target toxicity presents a problem for the effective use of (CAR)-T cells. To alleviate this, there is a need to produce off-tumor on-target toxicity.

**Figure 3:**
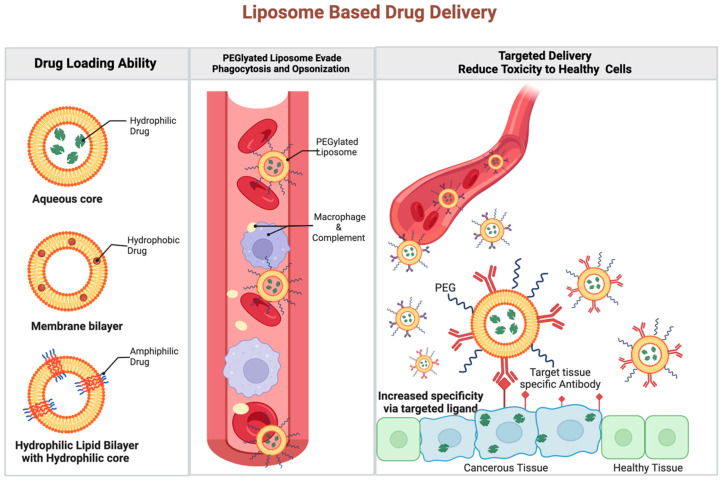
Liposome-based drug delivery. The lipid bilayer composition provides advantages in biocompatibility and the ability to encapsulate hydrophilic, lipophilic, and amphiphilic compounds to prevent their toxic effects. The highly modifiable surfaces of liposome vesicles can also be modified with tissue-specific antibodies for targeted delivery. Created with BioRender.com.

The previously established use of nanoparticles in a drug delivery system provides an excellent scaffold for this issue. Like (CAR)-T cell therapy, chemotherapy has faced many challenges in trying to avoid toxicity to healthy tissue. As an example, Tamoxifen citrate, an estrogen antagonist, showed great results in its ability to control breast cancer and prevent its relapse; however, the drug also has estrogen agonistic effects in other tissues such as the endometrium, increasing the risk of endometrial cancer [51]. Thus far, protein-based nano-formulations such as the albumin-bound form of paclitaxel, Abraxane, have been particularly helpful in influencing T cells to have better anti-tumoral immune responses due to their tendency to accumulate in tumors and improve aqueous solubility of hydrophobic drug compounds that may be used in conjunction with immunotherapy [73,74,75]. In the case of Abraxane, traditional paclitaxel holds a high risk of toxicity due to its promotion of microtubule polymerization and administration with the solvent Cremophor. Conjugation with an albumin-bound nanoparticle maintains the therapeutic effects of paclitaxel without the adverse effects of its typical solvent [75]. This allows for better penetration of existent T cells and promotes the patient’s own immune system to attack the tumor. Though this has not yet been used in conjunction with (CAR)-T cell therapy, there is potential for an effective synergistic effect. Liposome nanocarriers have also demonstrated great potential due to their lack of immunogenic components. This advantage has been exploited in anti-cancer drug delivery systems as well as the construction of (CAR)-T cells but has yet to be used in (CAR)-T cell targeting (Figure 3). These may be interesting paths for researchers to pursue in the future.

### 3.3. Downregulating Immunosuppression by the Tumor Microenvironment

One of the limitations of (CAR)-T cell therapy is the production of immunosuppressive molecules and the overexpression of immunosuppressive receptors like PD-L1. This can be a challenging obstacle, especially in solid tumors because the isolated environment of the tumor prevents the effective entry of the engineered T-cells. Nanotechnology provides a possible solution for inhibiting these immunosuppressive molecules or receptors. Hamilton et al. presented an ionizable lipid nanoparticle that not only suppresses the PD-1 pathway but does so locally without causing widespread immune suppression associated with antibody blockades. This is achieved by co-delivering CAR mRNA and PD-1-targeting siRNA to human T-cells using lipid nanoparticles [76]. On the other hand, Zhu et al. adopted a synergistic strategy by employing tumor-targeting nanozymes with photothermal-nanocatalytic properties to remodel the TME, ablate the tumor, and enhance (CAR)-T cell therapy effectiveness. This study utilized the tumor-specific HA@Cu2-xS-PEG (PHCN) nanozyme, comprised of hyaluronic acid (HA), as a targeting ligand and copper (Cu) for photothermal therapy (PTT). Copper serves as a good nanocatalyst due to its wide range of oxidation state and high near-infrared (NIR) absorption. Upon local exposure to NIR laser, activated copper converts light energy into heat, inducing apoptosis in nearby tumor cells. Additionally, copper catalyzes the local conversion of hydrogen peroxide into cytotoxic hydroxyl radicals, further promoting apoptosis. Following cell lysis, the release of tumor-specific antigens facilitates (CAR)-T cell activation. This ultimately improves (CAR)-T-cell infiltration and antitumor activity [77].

Limitations of (CAR)-T cell treatment of solid tumors are highly correlated with constraints of the TME. Nanotechnology has proven to be a possible path through which this therapy can better target these tumors, primarily by extending the exposure of the solid tumor to the targeting therapy. Grosskopf et al.’s simple injectable hydrogel that simultaneously delivers both (CAR)-T cells and stimulatory cytokines has demonstrated success in the treatment of solid tumors due to its avoidance of immunosuppression in the TME [78]. This material is created using a solution of dodecyl-modified hydroxypropyl methylcellulose (HPMC-C12) mixed with a biodegradable nanoparticle solution containing polyethylene glycol-b-polylactic acid (PEG-PLA NPs) [79]. The unique construction of this hydrogel allows for extended retention and activity of (CAR)-T cells in solid tumors which helps to overcome the ephemeral nature of targeting drugs to the vascular tumor environment [78]. This increased exposure of the patient to the therapy yields resultant improvement in therapy [74]. Gu et al. have also demonstrated success in the use of nanotechnology in conjunction with photothermal-therapy-aided (CAR)-T cell therapy. The wrapping of the near-infrared (NIR) dye indocyanine green (ICG) with polylactic-co-glycolic acid (PLGA) nanoparticles ensures stability of the photothermal agent as it is delivered into the solid tumor [3,80]. This, too, ensures extended exposure of the tumor to the targeting drug, thus allowing for more effective use of the original photothermal (CAR)-T cell therapy.

### 3.4. Nanobody-Based (CAR)-T Cells

All these ways in which nanotechnology has enhanced (CAR)-T cell therapy lead to one of the most interesting potential uses of nanotechnology in T-cell therapy: the construction of the CAR. As discussed in earlier sections, CARs have typically been based on a scFv that targets specific antigens on tumors. Recently, however, there have been studies of nanobody-based (CAR)-T cells that are as functional as their preceding scFv-based (CAR)-T cells in a preclinical as well as a clinical setting. These nanobodies, or variable regions of heavy-chain antibodies (VHHs), can be directed at any tumor antigens (VEGFR2, HER2, etc.) and are physiochemically stable [81,82]. The utilization of nanobodies was presented after the discovery of side effects like immune reactions against the linker or other domains in scFv [83]. Nanobodies have demonstrated poor immunogenicity in comparison [84]. scFvs have also illustrated CAR aggregation, leading to (CAR)-T exhaustion, one of the shortcomings of T cell therapy discussed previously [85]. Nanobody-based CARs have not shown CAR surface aggregation, reducing the limitation of T cell exhaustion [81].

Primarily, these nanobody-based CARs have been used to further increase the targeting of chosen tumor antigens. Mo et al. introduced CD105-specific nanobodies, also called anti-human CD105 (CAR)-T cells, as a potential therapy against CD105-positive tumors. It was created by using CRISPR-Cas9 technology to insert CD105 nanobody-linked standard cassette genes into the AAVS1 site. Subsequent inoculation of this with CD105-positive target cells allowed for the proliferation of anti-CD105 (CAR)-T cells. These T cells showed significant inhibition of implanted CD105+ tumors in mice and greater longevity of the implanted NOD/SCID mice [82]. D. Li et al. and H. Li et al. obtained similar findings with nanobodies targeting the IgC domain of B7-H3 in solid tumors and (HLA)-A2/GPC3 or HLA-A2/WT1 in MHC/peptide complex-expressing tumors, respectively [86,87]. 

The anti-HER2 nanobody has also been extensively studied due to 20–30% of breast cancer cases exhibiting HER2 overexpression [88]. Preclinical studies have demonstrated the efficacy of anti-HER2 nanobodies in interfering with HER2-mediated growth signaling pathways, such as the RAS-RAF-MAPK and PI3K-AKT-mTOR pathways, in addition to promoting tumor cytotoxicity through both antibody-dependent cellular cytotoxicity (ADCC) and complement-mediated immune activation [89]. Like other researched nanobodies, these combined effects result in decreased tumor growth, survival, and metastasis. Aside from its direct cytotoxic effects and growth inhibition, the anti-HER2 nanobody also shows unique promise as a positron emission tomography (PET) imaging probe for visualizing HER2-positive tumors. In an in vivo study using a mouse tumor model, the N-succinimidyl-4-[18F]fluorobenzoate-labeled anti-HER2 nanobodies exhibited high specific uptake into HER2-positive xenografts, producing high-contrast PET images [90]. Further development of the anti-HER2 nanobody may involve exploring synergistic combinations with other therapeutic modalities, such as Trastuzumab, to improve efficacy in trastuzumab-resistant cancer cells. Xavier et al. have shown that the anti-HER2 nanobody binds to and inhibits ligand-driven HER2-heterodimer formation, a mechanism contributing to Trastuzumab resistance, ultimately reducing tumor resistance [91].

These studies indicate that a variety of applications are available for the incorporation of nanotechnology into T cell therapies (Table 4). Further research into nanobody-based (CAR)-T cells with other suitable tumor targets (Table 2), particularly in solid tumors, may be especially worth pursuing.

## 4. Challenges of Incorporating Nanotherapy

The use of nanoparticles as carriers for targeted drug delivery, imaging agents, and chemoprevention has sparked interest in their ability to improve current treatment efficacy, minimize adverse effects, and improve prognosis. However, the application of nanomedicine from laboratory to clinic is accompanied by many obstacles, including manufacturing and commercial production, safety challenges and toxicity, and environmental risk. 

### 4.1. Challenges in Manufacturing and Regulating Nanoparticles

Nanoparticles are minute three-dimensional constructs with sizes typically ranging from 1 to 100 nanometers that exhibit unique behaviors due to their large surface area-to-volume ratio. They are often engineered with various shapes and surface compositions, allowing them to carry out specific functions [96]. This intricate structure of nanoparticles greatly complicates the manufacturing process since even subtle changes in the size, shape, and spatial arrangement of surface compositions can cause adverse effects or toxicity in the human body. For this reason, early development of nanoparticles usually takes place on a small scale, where the formulation process can be finely regulated to maintain ideal conditions for the synthesis and storage of nanoparticles [97]. However, to commercialize nanomedicine, its manufacturing must be up-scaled while maintaining precision and sterile conditions at every step of the process. Employing these highly technical processes comes with a high cost and a need for expertise, thus posing numerous economic challenges in addition to technical ones. 

A huge barrier to the commercialization of nanomedicine is overcoming the regulatory hurdles set by the FDA, European Medicines Agency, and other agencies. Since the implementation of nanomedicine is a relatively recent process, FDA regulation of nanoparticles is yet to be standardized, causing a lack of clarity and legal certainty. Not only does this impede the advancement of new nanotherapies into the market by complicating the process of obtaining FDA approval, it also raises concerns about the adequacy of regulatory oversight for existing nanoproducts [98]. To illustrate regulatory ambiguity in nanotherapy: particle size is not explicitly required to be disclosed by the applicant. The FDA may not realize that the product is nanotechnology-based until later in the regulatory process, contributing to delays in the process and insufficient governance. Unlike the traditional approval process for drugs with known active ingredients, nanoparticles, which most likely are either new molecular entities or are synthesized by manufacturers who are first-time applicants, will require pre-approval inspection. If the FDA is aware early on that the drug contains nanoparticles, it could schedule a pre-approval inspection of the facilities used for manufacture, to be more time efficient [99].

A great example of these challenges is exemplified by Feridex, also known as ferumoxides. Feridex is a magnetic resonance imaging (MRI) contrast agent containing superparamagnetic iron oxide nanoparticles. It was once extensively utilized for liver imaging, particularly in the detection and characterization of hepatocellular carcinoma and metastases [100]. However, it was largely discontinued and withdrawn from the market in 2008 due to its association with iron overload and severe hypersensitivity reactions in some patients [101]. Additionally, the manufacturing of Feridex may have become economically impossible for pharmaceutical companies. Issues such as manufacturing costs, supply chain logistics, and stricter regulatory requirements for the marketing of MRI contrast agents contributed to the decision to discontinue Feridex. These combined problems, along with a dwindling number of clinical users, ultimately led to Feridex being withdrawn from the market [102]. On the other hand, a successful example of nanoparticles is Doxil, the first FDA-approved nano-drug. Doxil is a liposome-encapsulated form of doxorubicin, allowing for prolonged circulation and specific targeting to tumor tissues through the enhanced permeability and retention (EPR) effect. After reaching the target tissue, doxorubicin is released to intercalate with DNA, inhibiting replication and transcription, ultimately leading to cancer cell death. In contrast to Feridex, Doxil has been widely used in clinical practice, particularly in the treatment of ovarian cancer and Kaposi’s sarcoma [103]. However, due to its liposomal technology and manufacturing costs, Doxil is more expensive than the generic doxorubicin.

### 4.2. Safety Challenges and Toxicity of Nanoparticles

Though there is a vast variety of nanoparticles, AuNPs are the most commonly used and studied. Previously in the discussion on employing AuNPs for cancer treatment, we highlighted their relatively low toxicity owing to their non-immunogenic nature as a noble metal and their ability to be secreted from the body through the renal system [104]. Given these advantages and coupled with FDA approval of AuNPs for various biomedical uses, a broader range of applications both within and beyond the medical field appears likely. These applications include cancer imaging and drug delivery as well as catalytic roles in carbon monoxide oxidation [105,106]. These increased applications of AuNP, however, can often be accompanied by prolonged exposures and accumulation of nanoparticles within cells to a toxic level [107].

Our review, which consolidates multiple in vitro and in vivo studies, needs to also consider the toxicological aspects of AuNPs. While some studies have shown that AuNPs are not toxic, many other studies contradict this statement. This review found that in some in vivo toxicity studies in which various AuNPs were injected into animal models (mostly mice and rats), a high accumulation of AuNPs was found in the liver, kidney, lung, and spleen, leading to oxidative tissue damage at the site (Table 5). In vitro toxicity studies further support this observation, showing that when hepatocytes were exposed to AuNPs, there was a dose and time-dependent increase in the production of reactive oxygen species (ROS). Additionally, outcomes of cell viability assays showed increases in cell mortality [108]. There is thus reason for concern that AuNPs, as well as other nanoparticles, might exhibit toxicity in some situations if used in human patients.

## 5. Conclusions

The integration of nanotechnology into (CAR)-T cell therapy emerges as a promising approach to overcome the limitations of (CAR)-T therapy highlighted in this review. Nanomedicine’s unique capabilities offer strategic advantages, including the blockade of immunosuppressive tumor environments, reduction of systemic toxicities, and stimulation of anti-tumor T cell responses. This overview of recent advancements, challenges, and potential strategies underscores the significant potential of nanotechnology to not only improve (CAR)-T cell immunotherapy but also to revolutionize immunotherapies at large. Our discussion aims to inspire further research and collaboration in this dynamic field, with the ultimate goal of making (CAR)-T cell therapy more accessible and effective against a wider array of cancers, particularly those solid tumors that have proven resistant to existing treatments. The potential of nanotechnology to transform T cell-based and broader immunotherapeutic approaches shines a light on the future of cancer treatment, urging a deeper exploration into this interdisciplinary area.

## Figures and Tables

**Table 4:** Nano-scaffold types and their applications to (CAR)-T cell immunotherapy [92,93,94,95].

Types	Examples	Application to (CAR)-T Cell Therapy
Polymeric	Poly(lactic-co-glycolic acid) (PLGA)	Carrier for T cells to enhance survival, increase uptake, and improve sustained drug release
Hydrogel	Hyaluronic acid	Carrier for enabling the continuous infusion of (CAR)-T cells, reducing sedimentation of the cells, and enhancing distribution
Lipid-based	Liposomes	Carrier for T cells to enhance survival
	Lipid Nanoparticles (LNPs)	Surface functionalization for targeting
Carbon-based	Carbon nanotubes (CNTs)	Provide mechanical strength High surface area for cargo loading The surface can be functionalized

**Table 5:** Results of AuNP exposure in both in vivo and in vitro studies.

Organism	Particle	Effects
Mice	AuNPs	AuNPs accumulate in spleen, liver, and lungs [109]
Rats	AuNPs	AuNPs accumulate in spleen and liver [110]
Mice	PEG-coated AuNPs	Apoptosis and acute inflammation [111]
Rats	PEG-coated AuNPs	Accumulation in spleen and liver [111]
Human liver cells	AuNPs	Depolarization of mitochondrial transmembrane potential and apoptosis [112]
Airway epithelial cells	AuNPs	Elevated lipid peroxidase and DNA damage [113]
